# Fertility preservation in girls and adolescents with Turner syndrome: lessons for reproductive care in differences of sex development - a mini-review

**DOI:** 10.3389/fendo.2026.1918808

**Published:** 2026-09-30

**Authors:** Zofia Kolesińska, Monika Obara-Moszyńska, Marek Niedziela, Karina Kapczuk

**Affiliations:** 1 Department of Pediatric Endocrinology and Rheumatology, Institute of Pediatrics, Poznan University of Medical Sciences, Poznań, Poland; 2 Division of Gynecology, Unit of Pediatric and Adolescent Gynecology, Poznan University of Medical Sciences, Poznań, Poland

**Keywords:** differences (disorders) of sex development, fertility preservation, oocyte cryopreservation, ovarian tissue cryopreservation (OTC), Turner syndrome

## Abstract

Current knowledge regarding fertility preservation (FP) in girls is derived predominantly from pediatric oncology cohorts; however, FP is increasingly being applied to non-oncological conditions associated with impaired fertility, including differences of sex development (DSD). Turner syndrome (TS), characterized by accelerated germ cell depletion beginning during fetal life, represents the DSD condition with the greatest clinical experience in FP. This mini-review summarizes current evidence on ovarian tissue cryopreservation and oocyte cryopreservation in TS. Available data support the technical feasibility and general procedural safety of both approaches. A 46,XX cell line, spontaneous pubertal development, detectable AMH, and lower FSH concentrations are frequently associated with follicle detection or successful oocyte retrieval. However, evidence regarding utilization, pregnancy, and live birth remains very limited, and the reproductive effectiveness of FP in TS has not yet been established. Moreover, future cardiovascular eligibility for pregnancy and associated obstetric risks should be considered when counseling patients and families. Until long-term outcome data become available, individualized patient selection and multidisciplinary counseling are essential to provide realistic expectations regarding the potential benefits, limitations, and uncertainties of FP. Experience in TS may also inform reproductive care in other DSD conditions.

## Introduction

Advances in reproductive medicine have expanded fertility preservation (FP) options for girls and adolescents at risk of premature ovarian insufficiency (POI). In 2012, the American Society for Reproductive Medicine (ASRM) and the European Society of Human Reproduction and Embryology (ESHRE) recognized oocyte cryopreservation (OC) as a standard FP option (1, 2). Subsequently, in 2019, ASRM concluded that ovarian tissue cryopreservation (OTC) should no longer be considered experimental and acknowledged it as an established clinical procedure for patients undergoing gonadotoxic therapy, including children and adolescents (3). Currently, OTC remains the only available FP strategy for prepubertal girls, as mature oocyte retrieval is not feasible before puberty. OTC has become increasingly integrated into clinical practice, resulting in more than 200 live births worldwide following transplantation of cryopreserved ovarian tissue, predominantly in adult women, by 2020 (4, 5).

Although FP was initially developed for patients undergoing gonadotoxic therapy, interest has increasingly expanded to non-oncological conditions associated with impaired fertility. Turner syndrome (TS), in which accelerated depletion of the ovarian follicle pool frequently results in loss of reproductive potential before adulthood, has emerged as a major indication for FP in this setting (6). Fertility counseling is increasingly recognized as an essential component of care in individuals with differences of sex development (DSD), as reproductive potential and parenthood represent important long-term outcomes affecting quality of life (7–9). However, FP in DSD poses unique challenges related to gonadal development, progressive germ cell loss, gonadal malignancy risk, reproductive anatomy, and genetic transmission (10). Most of the available evidence is derived from studies of girls and adolescents with TS. This review summarizes current evidence regarding the feasibility, safety, and potential reproductive benefit of FP in girls and adolescents with TS.

## Methodology

To identify reports on FP in girls and adolescents with TS, we searched Ovid MEDLINE, Embase, Web of Science, and Google Scholar between January 1990 and June 2026. Search terms were adapted to each database (**Supplementary Material**). Reference lists of relevant publications were also screened to identify additional eligible studies. Studies reporting OTC or OC in girls and adolescents with TS were included. Where studies reported overlapping or potentially overlapping cohorts, this was considered when interpreting cumulative patient numbers; however, overlap could not always be excluded. Accordingly, pooled numerators and denominators are presented as descriptive estimates.

## Lessons from pediatric oncology fertility preservation programs

Current clinical experience with FP in girls is derived predominantly from pediatric oncology. Although the clinical context differs from TS, as FP is primarily offered to girls with malignant diseases before exposure to gonadotoxic treatment, this experience provides valuable evidence regarding the scale of clinical implementation, procedural feasibility, safety, and long-term outcomes.

Since the first reported pediatric FP procedures in 1993, approximately 2,500 girls and adolescents have undergone FP procedures before gonadotoxic treatment. The majority underwent OTC, commonly by unilateral oophorectomy, whereas OC has increasingly been performed in postpubertal adolescents (11–13) (**Supplementary Table 1**). Both procedures appear technically feasible and generally safe, with complications reported only rarely and predominantly limited to moderate postoperative bleeding following OTC (14) and moderate ovarian hyperstimulation syndrome (OHSS) after OC (15–17) (**Supplementary Table 1**).

Data on long-term utilization, however, remains limited (**Supplementary Table 2**). Explicit return rates following childhood FP have been reported in only one study, with no returns among children and a return rate of only 1% among adolescents (18). Only 18 ovarian tissue transplantations have been reported; although ovarian endocrine function was restored in most evaluable patients, only six achieved at least one live birth (LB) (**Supplementary Table 2**). Evidence regarding oocytes cryopreserved during adolescence is even more limited, with only a single reported LB (19).

## Fertility preservation in the context of differences of sex development

FP has become an increasingly important component of comprehensive care for individuals with DSD (7–9). Reproductive potential varies considerably between conditions, and discussions and decisions regarding FP may be complicated by differences in gonadal development, progressive germ cell loss, malignancy risk, reproductive anatomy, associated comorbidities, and genetic transmission (10, 20). As evidence regarding reproductive outcomes remains scarce, counseling should emphasize the experimental nature and uncertain long-term benefit of FP and involve age-appropriate, multidisciplinary shared decision-making with patients and families (20, 21). Among DSD conditions, the greatest clinical experience with FP has been accumulated in TS. Survey data consistently demonstrate a strong desire for parenthood among individuals with TS (22), and 25-73% of patients referred for fertility counseling ultimately underwent a FP procedure (23–25). Nevertheless, TS also presents several condition specific challenges that distinguish it from both oncology and other DSD conditions.

## Pathophysiological basis for fertility preservation in Turner syndrome

Unlike cancer survivors, girls with TS undergo FP not to protect a normal ovarian reserve from gonadotoxic treatment, but to preserve the remaining follicular pool undergoing premature depletion. Most women with TS develop POI as a consequence of accelerated germ cell loss, which begins during fetal life (26). Although primordial germ cell migration and early gonadal colonization appear relatively preserved (6, 27, 28), a marked reduction in OCT4-postive oogonia becomes evident from approximately 13–14 gestational weeks and is accompanied by increased apoptosis (28). Extensive apoptosis of primary oocytes subsequently occurs, resulting in markedly impaired primordial follicle formation (29) and exceeding physiological prenatal oocyte atresia.

The mechanisms underlying this accelerated depletion remain incompletely understood and are likely multifactorial. Because germ cell loss becomes evident around the time of meiotic entry, abnormal meiotic progression related to X-chromosome monosomy has long been implicated. However, ultrastructural studies indicate that many germ cells fail to progress beyond the earliest stages of meiotic prophase, suggesting that defective homologous chromosome pairing alone cannot explain ovarian failure (30). More recent evidence indicates that X-chromosome dosage within the oocyte itself may be particularly important for follicular survival. In mosaic TS ovaries, preserved follicles predominantly contain euploid 46,XX oocytes, whereas the surrounding granulosa cells may frequently be 45,X (31, 32).

Importantly, the presence of a 46,XX oocyte does not necessarily imply normal follicular function. Oocyte development depends on bidirectional signaling with the surrounding granulosa cells and the stromal microenvironment. Transcriptomic studies have demonstrated altered gene expression in small follicles from girls with mosaic TS, suggesting that X-chromosome monosomy in somatic ovarian cells may impair oocyte-somatic cell interactions and contribute to progressive follicular depletion (33). These findings support the concept that impaired oocyte–somatic cell communication may contribute to progressive follicular depletion in TS. Finally, haploinsufficiency of X-linked genes may also contribute to ovarian dysfunction, as reduced expression of genes escaping X-chromosome has been implicated in ovarian failure (34). Candidate genes include *BMP15* and *PGRMC1*, which play important roles in oocyte maturation, granulosa cell function and folliculogenesis (35, 36).

More recent single–cell transcriptomic studies of human fetal ovaries provide a broader perspective on these mechanisms. Perimeiotic 45,X ovaries showed reduced germ cell numbers across all germ cell subpopulations, depletion of an oogonial population expressing genes involved in sex-chromosome synapsis, disruption of the normal sequence of X-chromosome inactivation and reactivation, and widespread transcriptional abnormalities involving pathways related to proteostasis, cell-cycle progression and oxidative phosphorylation (37). These findings suggest that germ cell depletion in TS may reflect a broader disruption of the molecular program of ovarian and germ cell development rather than a defect in a single meiotic pathway or individual X-linked gene.

## Predictors of presence of primordial follicles

Although POI is frequent in TS, residual ovarian function persists in a subset of patients, with spontaneous puberty onset occurring in approximately 30%, menarche in 15%, and spontaneous pregnancy (SP) in around 5% (38, 39). This indicates that accelerated follicular depletion continues after birth and may provide a window for FP before complete exhaustion of the ovarian reserve.

Mosaic karyotype, spontaneous puberty, lower FSH, and detectable AMH concentrations have been identified as predictors of residual primordial follicles (40, 41). However, none of these parameters, either alone or in combination, can definitively exclude the presence of follicles. Primordial follicles have been identified in girls with an apparently non-mosaic 45,X karyotype and unfavorable hormonal markers (40–42), while SP have also been reported in women classified as 45,X, potentially reflecting undetected tissue-specific or cryptic mosaicism (43–46). Assessment of ovarian potential should therefore integrate cytogenetic, clinical and biochemical markers while recognizing their limitations.

## Pregnancy in Turner syndrome is considered high risk

Counseling regarding FP in TS should extend beyond the possibility of preserving ovarian tissue or oocytes to consider whether future pregnancy will be medically appropriate. Pregnancy in women with TS is considered high risk, primarily because of cardiovascular complications, which remain a major contributor to the increased mortality observed in this population (47).

One of the first attempts to quantify the risk of maternal mortality and aortic dissection (AD) during pregnancy combined a questionnaire survey of pregnancies achieved through oocyte donation (OD) with a literature review. Based on the reported cases and extrapolation of the available data, the risk of maternal mortality and AD was estimated at approximately 2%, corresponding to a 100–200-fold higher risk than in the general female population (48). Subsequent studies identified major risk factors for AD, including bicuspid aortic valve, coarctation of the aorta, dilatation of the ascending aorta, and arterial hypertension (49), although AD may occasionally occur in the absence of recognized cardiovascular risk factors.

These observations led to recommendations for comprehensive cardiovascular assessment before conception and close surveillance throughout pregnancy and the postpartum period (50–52). Reassuringly, with appropriate preconception screening and intensive surveillance, more recent data suggest that the incidence of AD may be substantially lower than initially estimated at approximately 0.5% (26, 53, 54). Notably, maternal deaths following AD reported to date have predominantly occurred after OD, whereas none have been reported following SP (39, 55–59).

Although AD represents the most life-threatening-pregnancy-related complication in TS, reproductive and obstetric risks extend beyond cardiovascular morbidity. Following spontaneous conception or *in vitro* fertilization with autologous oocytes (IVF-AO), miscarriage occurs in approximately 45% of pregnancies (39, 60), while hypertensive disorders of pregnancy have been reported in up to 13% (61, 62). Adverse fetal and neonatal outcomes include risks of small for gestational age and low birth weight (57, 62). Notably, these complications are even more frequent in pregnancies after OD. Finally, pregnancies achieved using AO carry a risk of chromosomal abnormalities in the offspring, particularly in women with structural X-chromosome abnormalities (61).

## Ovarian tissue cryopreservation in Turner syndrome

Published studies collectively describe approximately 220 girls and adolescents with TS who underwent OTC, although overlap between some reported cohorts cannot be excluded (**Table 1**; **Supplementary Table 1**). The median age at cryopreservation was 13.5 years (range 3.0–17.8 years). Ovarian tissue was obtained in nearly all patients, with only a small number of procedures failing because of streak gonads (41). Histological evaluation identified follicles in approximately 43.7% (83/190) of evaluable patients.

**Table 1:** Clinical characteristics of girls and adolescents with Turner syndrome undergoing ovarian tissue cryopreservation.

No.	Reference	Patients (N)	Age at OCT [yrs]	Ovarian tissue detected (n/N)	Follicles detected (n/N)	Patients with detected follicles (N)							
						karyotype (n/N)					SP (n/N)	detectable AMH (n/N)	FSH ≤ 10 (n/N)
						45,X	with 46,XX line	45,X/ 47,XXX	45,X/ SA	SA			
1	Hreinsson et al., 2022 (40)	9	median 15 (range 12–17)	8/9	5/8	2/5	3/5	0/5	0/5	0/5	5/5	N/R	5/5
2	Huang et al., 2008^1^ (66)	1	16	1/1	1/1	0/1	1/1	0/1	0/1	0/1	1/1	N/R	1/1
3	Borgstrom et al., 2009 (41)	48	median 13.8 (range 8–17.8)	38/48	13/38	2/13	6/13	0/13	2/13	3/13	10/12	12/12	9/13
4	Balen et al., 2009 (67)	1	17	1/1	1/1	0/1	1/1	0/1	0/1	0/1	1/1	N/R	1/1
5	Mamsen et al., 2019 (68)	12	median 14.55 (range 5–17.8)	12/12	7/12	1/7	4/7	0/7	2/7	0/7	5/5	5/5	5/5
6	Peek et al., 2019 (31)	10	median 13.5 (range 3–17)	10/10	5/10	0/5	5/5	0/5	0/5	0/5	N/R	N/R	N/R
7	Cheng et al., 2022 (69)	1	3	1/1	1/1	0/1	1/1	0/1	0/1	0/1	N/A	1/1	1/1
8	Dunlop et al., 2023 (70)	1	15	1/1	1/1	0/1	0/1	0/1	1/1	0/1	1/1	1/1	1/1
9	Nadesapillai et al., 2023 and van der Coelen et al., 2024 (64, 65)	93	mean 11.9 (range 3–19)	93/93	30/93	1/28	21/28	1/28	3/28	2/28	16/18	27/28	28/28
10	Peek et al., 2023 and Schleedoorn et al., 2022 (32, 42)	17^2^	median 12 (range 5–17)	17/17	17/17	1/17	14/17	2/17	0/17	0/17	10/10	15/15	16/17
11	Rodriguez-Wallberg et al., 2023 (24)	66^3^	N/D	as reported by Borgstrom et al., 2009 (41)									
12	Miyamoto et al., 2025 (71)	1	6	1/1	0/1	N/A	N/A	N/A	N/A	N/A	N/A	N/A	N/A
13	Sage et al., 2026 (23)	7	median 13 (range 3–7)	7/7	2/7	0/2	2/2	0/2	0/2	0/2	2/2	2/2	2/2
	**total**	**219**	**median 13.5 (range 3–17.8)^4^**	**190/201**	**83/190**	**7/81**	**58/81**	**3/81**	**8/81**	**5/81**	**51/55**	**63/64**	**69/74**

No., study number; OCT, ovarian tissue cryopreservation; SP, spontaneous puberty; SA, structural aberration; N/R, not reported; N/A, not applicable.

^1^performed concomitantly with oocyte cryopreservation; ^2^some overlap with the cohort reported by Peek et al., 2019 (31) cannot be excluded; ^3^48 cases already included in study by Borgstrom et al., 2009 (41); ^4^calculated excluding the cohort reported by Nadesapillai et al., 2023 (64).

Bold values indicate pooled numerators and denominators across studies.

Among girls in whom follicles were detected, 71.6% (58/81) had a 46,XX cell line, 92.7% (51/55) had spontaneous puberty, 98.4% (63/64) had detectable AMH levels, and 93.2% (69/74) had FSH concentrations ≤10 IU/L. Conversely, follicles were also identified in seven girls classified as 45,X (8.6%; 7/81), four without spontaneous puberty (7.3%; 4/55), one with undetectable AMH (1.6%; 1/64), and five girls with FSH >10 IU/L (6.8%; 5/74) (**Table 1**). These descriptive data indicate that a 46,XX cell line, spontaneous puberty, detectable AMH, and lower FSH are characteristics frequently observed among girls with detected follicles. Although AMH is considered a limited marker of ovarian reserve in pediatric population (63), available evidence suggests that detectable AMH may be a useful indicator of the presence of follicles in girls with TS.

Only one study reported complications related to OCT (64); these included minor procedural complications and psychological distress related to the absence of follicles in the cryopreserved tissue, including one report of suicidal ideation. Evidence regarding the effect of unilateral oophorectomy on residual ovarian function is limited to a single study. Pubertal development continued after the procedure in most girls, whereas those with reduced ovarian reserve before OTC were likely to require hormone replacement therapy within the following years (65).

## Oocyte cryopreservation in Turner syndrome

Data are currently available for 31 girls and adolescents with TS who underwent OC before the age of 18 years (**Table 2**). The median age at cryopreservation was 15 years (range 7.0–17.0 years), although the procedure has been reported in a prepubertal girl as young as 7 years of age (72). Mature oocytes were successfully cryopreserved in 80.6% of patients (25/31). Successful OC was observed predominantly in patients with a 46,XX cell line (52.0%; 13/25), spontaneous puberty (91.3%; 21/23), detectable AMH levels (93.3%; 14/15), and FSH concentrations ≤10 IU/L (100.0%; 23/23). No complications, including OHSS, were reported (**Table 2**).

**Table 2:** Clinical characteristics of girls and adolescents with Turner syndrome undergoing oocyte cryopreservation.

No.	Reference	Age at OC [yrs]	COH (n)	Oocytes retrieved (n)	Oocytes cryopreserved (n)	Karyotype (n)					SP (n)	Detectable AMH (n)	FSH ≤ 10 (n)
						45,X	with 46,XX line	45,X/ 47,XXX	45,X/SA	SA			
1	Huang et al., 2008^1^ (66)	16	IVM	11	8	0	1	0	0	0	1	N/R	1
2	Lau et al., 2009 (73)	16	1	2	2	0	0	1	0	0	1	N/R	1
3	Oktay et al., 2010 (74)	14 and 15	2	18	18^2^	0	1	0	0	0	1	1	1
4	Oktay and Bedoschi, 2014 (75)	13	1 + IVM	19	10	0	0	1	0	0	1	1	1
5	Oktay and Bedoschi, 2014 (75)	13	1 + IVM	16	12	0	1	0	0	0	1	1	1
6	Azem et al., 2020 (72)	7	2	6	6	0	0	1	0	0	N/A	1	1
7	Martel et al., 2022 (76)	13	1	14	14^2^	0	1	0	0	0	1	1	1
8	Martel et al., 2022 (76)	14	1	4	4^2^	1	0	0	0	0	0	0	1
9	Martel et al., 2022 (76)	14	1	21	21^2^	0	1	0	0	0	1	1	1
10	Martel et al., 2022 (76)	14	3	0	0	1	0	0	0	0	1	1	0
11	Martel et al., 2022 (76)	15	2	0	0	1	0	0	0	0	N/R	N/R	1
12	Martel et al., 2022 (76)	15	1	15	15	1	0	0	0	0	N/R	N/R	1
13	Martel et al., 2022 (76)	15	1	0	0	0	1	0	0	0	1	0	1
14	Martel et al., 2022 (76)	16	3	19	18^2^	1	0	0	0	0	1	1	1
15	Martel et al., 2022 (76)	17	9	3	3^2^	0	1	0	0	0	1	N/R	1
16	Brouillet et al., 2023 (25)	17	1	11	11	0	1	0	0	0	1	1	1
17	Brouillet et al., 2023 (25)	16	2	24	20	0	1	0	0	0	0	1	1
18	Brouillet et al., 2023 (25)	16	3	2	1	0	0	0	1	0	1	1	1
19	Brouillet et al., 2023 (25)	15	1	0	0	0	0	1	0	0	1	1	0
20	Brouillet et al., 2023 (25)	17	1	5	5	0	1	0	0	0	1	1	1
21	Nadesapillali et al., 2023 (77)	17	1	N/R	31	0	1	0	0	0	1	N/R	1
22	Nadesapillali et al., 2023 (77)	17	2	N/R	19	0	1	0	0	0	1	N/R	1
23	Nadesapillali et al., 2023 (77)	17	3	N/R	9	0	0	0	0	1	1	N/R	1
24	Nadesapillali et al., 2023 (77)	17	2	N/R	26	0	0	0	1	0	1	N/R	N/R
25	Nadesapillali et al., 2023 (77)	17	1	N/R	5	0	0	0	0	1	1	N/R	N/R
26	Nadesapillali et al., 2023 (77)	16	1	N/R	1	0	0	1	0	0	1	N/R	1
27	Nadesapillali et al., 2023 (77)	17	1	N/R	0	0	0	1	0	0	1	N/R	0
28	Beroukhim et al., 2025 (78)	14	2	0	0	0	0	0	1	0	0	N/R	N/R
29	Beroukhim et al., 2025 (78)	15	1	N/R	16	0	1	0	0	0	1	1	1
30	Beroukhim et al., 2025 (78)	13	1	N/R	5	0	1	0	0	0	1	1	1
31	Beroukhim et al., 2025 (78)	14	1	N/R	3	0	0	1	0	0	1	1	1
	**total**	**median 15 (range 7-17)**				**5**	**14**	**7**	**3**	**2**	**25**	**16**	**25**

No., number of patients; OC, oocyte cryopreservation; COH, controlled ovarian hyperstimulation; SP, spontaneous puberty; IVM, *in vitro* maturation; N/R, not reported; N/A, not applicable.

^1^performed concomitantly with ovarian tissue cryopreservation; ^2^including immature oocytes.

Bold values indicate pooled numerators and denominators across studies.

## Return, utilization, and reproductive effectiveness

Only three women with TS have been reported to return for attempted fertility restoration using ovarian tissue cryopreserved during adolescence (**Table 1**; **Supplementary Table 2**). In one, ovarian function was restored following transplantation and SP occurred within nine months but ended in miscarriage (70). In the remaining two women, ovarian function was not restored following transplantation (24). To date, no pregnancies or LB have been reported in women with TS using oocytes cryopreserved during adolescence.

## Ongoing prospective studies

Recognizing the need for long-term outcome data, two major prospective studies have been established. The Dutch TurnerFertility Trial (NCT03381300) is designed to evaluate LB rates following OTC during childhood and subsequent autotransplantation in adulthood. In addition, the study aims to identify reliable prognostic markers of ovarian reserve and reproductive potential in girls with TS (79). Recruitment and OCT procedures were completed in December 2021, and follow-up is ongoing, with study completion anticipated in 2071. The Danish Turner Syndrome Cryopreservation Study (NCT05740579) is currently recruiting participants. In addition to evaluating long-term reproductive outcomes, the study aims to identify early predictors of POI and improve understanding of ovarian dysgenesis in TS. Follow-up is expected to continue until 2050 (80).

## Conclusions

Current evidence supports the technical feasibility and general procedural safety of OTC and OC in girls and adolescents with TS. A 46,XX cell line, spontaneous pubertal development, detectable AMH, and lower FSH concentrations are frequently associated with follicle detection or successful oocyte retrieval. Importantly, evidence regarding utilization, pregnancy, and LB remains extremely limited, and the reproductive effectiveness of FP in TS has not yet been established.

Patient selection and counseling should therefore address not only residual ovarian reserve, but also the possibility of unsuccessful follicle retrieval, uncertain oocyte competence, potential effects of ovarian surgery, future cardiovascular eligibility for pregnancy, obstetric and genetic risks, and the possibility that cryopreserved material may never be used. Particular attention should be given to avoiding unrealistic expectations and associated psychological distress.

Ongoing prospective studies will be essential to determine the long-term effectiveness of FP strategies in TS. As most of the current evidence regarding FP in DSD has been generated in girls and adolescents with TS, insights gained from this population may help inform future reproductive care and FP approaches in other DSD conditions. Until such data become available, FP should be offered within a multidisciplinary framework that provides realistic counseling regarding potential benefits, limitations, and uncertainties.
