# Supplementary material for: Fertility preservation in girls and adolescents with Turner syndrome: lessons for reproductive care in differences of sex development - a mini-review
Source: Front Endocrinol (Lausanne). 2026 Sep 30;17:1918808. doi: 10.3389/fendo.2026.1918808 (PMC13662555; doi:10.3389/fendo.2026.1918808)
Supplement: Supplementary file 1 [file SupplementaryFile1.docx]

**Supplementary material:**

**Search strategy:**

**Embase search terms:**

(("Fertility preservation" OR "fertility preservation" OR "Ovarian tissue cryopreservation" OR "ovarian tissue cryopreservation" OR "Oocyte cryopreservation" OR "oocyte cryopreservation") AND ("Turner Syndrome" OR TS OR "Ullrich-Turner syndrome" OR "Ullrich Turner syndrome" OR "Turners syndrome" OR "45,X gonadal dysgenesis" OR "XO gonadal dysgenesis" OR "monosomy X" OR "Bonnevie-Ullrich syndrome" OR "Bonnevie Ullrich syndrome"))

**Google Scholar search terms:**

((fertility preservation OR ovarian tissue cryopreservation OR oocyte cryopreservation) AND (Turner Syndrome OR monosomy X))

**Web of Science search terms:**

TS=((Fertility preservation OR fertility preservation OR Ovarian tissue cryopreservation OR ovarian tissue cryopreservation OR Oocyte cryopreservation OR oocyte cryopreservation) AND ("Turner Syndrome" OR TS OR "Ullrich-Turner syndrome" OR "Ullrich Turner syndrome" OR "Turner’s syndrome" OR "Turners syndrome" OR "45,X gonadal dysgenesis" OR "XO gonadal dysgenesis" OR "monosomy X" OR "Bonnevie-Ullrich syndrome" OR "Bonnevie Ullrich syndrome"))

**Ovid MEDLINE search terms:**

(("Fertility preservation"[Mesh] OR "fertility preservation"[tw] OR "Ovarian tissue cryopreservation"[Mesh] OR "ovarian tissue cryopreservation"[tw] OR "Oocyte cryopreservation"[Mesh] OR "oocyte cryopreservation"[tw]) AND ("Turner Syndrome"[Mesh] OR "Turner Syndrome"[tw] OR TS[tw] OR "Ullrich-Turner syndrome"[tw] OR "Ullrich Turner syndrome"[tw] OR "Turner’s syndrome"[tw] OR "Turners syndrome"[tw] OR "45,X gonadal dysgenesis"[tw] OR "XO gonadal dysgenesis"[tw] OR "monosomy X"[tw] OR "Bonnevie-Ullrich syndrome"[tw] OR "Bonnevie Ullrich syndrome"[tw]))

**References cited in Supplementary Tables 1 and 2:**

1.	Poirot C, Vacher-Lavenu MC, Helardot P, Guibert J, Brugières L, Jouannet P. Human ovarian tissue cryopreservation: indications and feasibility. Human Reproduction. 2002.

2.	Schmidt KLT, Byskov AG, Andersen AN, Müller J, Andersen CY. Density and distribution of primordial follicles in single pieces of cortex from 21 patients and in individual pieces of cortex from three entire human ovaries. Human Reproduction. 2003;18(6):1158–64. doi:10.1093/humrep/deg246 PubMed PMID: 12773440.

3.	Porcu E, Fabbri R, Damiano G, Fratto R, Giunchi S, Venturoli S. Oocyte cryopreservation in oncological patients. European Journal of Obstetrics and Gynecology and Reproductive Biology. 2004 Apr 5;113(SUPPL.). doi:10.1016/j.ejogrb.2003.11.004 PubMed PMID: 15041124.

4.	Bath LE, Tydeman G, Critchley HOD, Anderson RA, Baird DT, Wallace WHB. Spontaneous conception in a young woman who had ovarian cortical tissue cryopreserved before chemotherapy and radiotherapy for a Ewing’s sarcoma of the pelvis: Case report. Human Reproduction. 2004;19(11):2569–72. doi:10.1093/humrep/deh472 PubMed PMID: 15310731.

5.	Feigin E, Abir R, Fisch B, Kravarusic D, Steinberg R, Nitke S, et al. Laparoscopic ovarian tissue preservation in young patients at risk for ovarian failure as a result of chemotherapy/irradiation for primary malignancy. J Pediatr Surg. 2007 Jul;42(5):862–4. doi:10.1016/j.jpedsurg.2006.12.041 PubMed PMID: 17502200.

6.	Poirot CJ, Martelli H, Genestie C, Golmard JL, Valteau-Couanet D, Helardot P, et al. Feasibility of ovarian tissue cryopreservation for prepubertal females with cancer. Pediatr Blood Cancer. 2007 Jul;49(1):74–8. doi:10.1002/pbc.21027 PubMed PMID: 16977608.

7.	Anderson RA, Wallace WHB, Baird DT. Ovarian cryopreservation for fertility preservation: Indications and outcomes. Reproduction. 2008. p. 681–9. doi:10.1530/REP-08-0097 PubMed PMID: 18682546.

8.	Revel A, Revel-Vilk S, Aizenman E, Porat-Katz A, Safran A, Ben-meir A, et al. At what age can human oocytes be obtained? Fertil Steril. 2009 Aug;92(2):458–63. doi:10.1016/j.fertnstert.2008.07.013 PubMed PMID: 18952208.

9.	Oktay K, Oktem O. Fertility preservation medicine: A new field in the care of young cancer survivors. In: Pediatric Blood and Cancer. 2009. p. 267–73. doi:10.1002/pbc.22003 PubMed PMID: 19301406.

10.	Jadoul P, Dolmans MM, Donnez J. Fertility preservation in girls during childhood: Is it feasible, efficient and safe and to whom should it be proposed? Hum Reprod Update. 2010 May 12;16(6):617–30. doi:10.1093/humupd/dmq010 PubMed PMID: 20462941.

11.	Oktay K, Oktem O. Ovarian cryopreservation and transplantation for fertility preservation for medical indications: report of an ongoing experience. Fertil Steril. 2010 Feb;93(3):762–8. doi:10.1016/j.fertnstert.2008.10.006 PubMed PMID: 19013568.

12.	Donnez J, Squifflet J, Jadoul P, Demylle D, Cheron AC, Van Langendonckt A, et al. Pregnancy and live birth after autotransplantation of frozen-thawed ovarian tissue in a patient with metastatic disease undergoing chemotherapy and hematopoietic stem cell transplantation. Fertil Steril. 2011;95(5):1787.e1-1787.e4. doi:10.1016/j.fertnstert.2010.11.041

13.	Kim TJ, Hong SW. Successful live birth from vitrified oocytes after 5 years of cryopreservation. J Assist Reprod Genet. 2011 Jan;28(1):73–6. doi:10.1007/s10815-010-9487-3 PubMed PMID: 20886366.

14.	Schmidt KT, Rosendahl M, Ernst E, Loft A, Andersen AN, Dueholm M, et al. Autotransplantation of cryopreserved ovarian tissue in 12 women with chemotherapy-induced premature ovarian failure: The Danish experience. Fertil Steril. 2011 Feb;95(2):695–701. doi:10.1016/j.fertnstert.2010.07.1080 PubMed PMID: 20828687.

15.	Rosendahl M, Schmidt KT, Ernst E, Rasmussen PE, Loft A, Byskov AG, et al. Cryopreservation of ovarian tissue for a decade in Denmark: A view of the technique. Reprod Biomed Online. 2011 Feb;22(2):162–71. doi:10.1016/j.rbmo.2010.10.015 PubMed PMID: 21239230.

16.	Gracia CR, Chang J, Kondapalli L, Prewitt M, Carlson CA, Mattei P, et al. Ovarian tissue cryopreservation for fertility preservation in cancer patients: Successful establishment and feasibility of a multidisciplinary collaboration. J Assist Reprod Genet. 2012 Jun;29(6):495–502. doi:10.1007/s10815-012-9753-7 PubMed PMID: 22466745.

17.	Poirot C, Abirached F, Prades M, Coussieu C, Bernaudin F, Piver P. Induction of puberty by autograft of cryopreserved ovarian tissue. The Lancet. 2012;379(9815):588. doi:10.1016/S0140-6736(11)61781-9 PubMed PMID: 22325664.

18.	Babayev SN, Arslan E, Kogan S, Moy F, Oktay K. Evaluation of ovarian and testicular tissue cryopreservation in children undergoing gonadotoxic therapies. J Assist Reprod Genet. 2013 Jan;30(1):3–9. doi:10.1007/s10815-012-9909-5 PubMed PMID: 23242649.

19.	Huser M, Zakova J, Smardova L, Janku P, Hudecek R, Ventruba P. Combination of fertility preservation strategies in young women with recently diagnosed cancer.

20.	Reichman DE, Davis OK, Zaninovic N, Rosenwaks Z, Goldschlag DE. Fertility preservation using controlled ovarian hyperstimulation and oocyte cryopreservation in a premenarcheal female with myelodysplastic syndrome. Fertil Steril. 2012 Nov;98(5):1225–8. doi:10.1016/j.fertnstert.2012.07.1056 PubMed PMID: 22884018.

21.	Ernst E, Kjærsgaard M, Birkebæk NH, Clausen N, Andersen CY. Case report: Stimulation of puberty in a girl with chemo- and radiation therapy induced ovarian failure by transplantation of a small part of her frozen/thawed ovarian tissue. Eur J Cancer. 2013 Mar;49(4):911–4. doi:10.1016/j.ejca.2012.09.028 PubMed PMID: 23084082.

22.	Greve T, Schmidt KT, Kristensen SG, Ernst E, Andersen CY. Evaluation of the ovarian reserve in women transplanted with frozen and thawed ovarian cortical tissue. Fertil Steril. 2012;97(6). doi:10.1016/j.fertnstert.2012.02.036

23.	Rodriguez-Wallberg KA, Tanbo T, Tinkanen H, Thurin-Kjellberg A, Nedstrand E, Kitlinski ML, et al. Ovarian tissue cryopreservation and transplantation among alternatives for fertility preservation in the Nordic countries – compilation of 20 years of multicenter experience. Acta Obstet Gynecol Scand. 2016 Sep 1;95(9):1015–26. doi:10.1111/aogs.12934 PubMed PMID: 27258933.

24.	Dolmans MM, von Wolff M, Poirot C, Diaz-Garcia C, Cacciottola L, Boissel N, et al. Transplantation of cryopreserved ovarian tissue in a series of 285 women: a review of five leading European centers. Fertil Steril. 2021;115(5):1102–15. doi:10.1016/j.fertnstert.2021.03.008 PubMed PMID: 33933173.

25.	Hornshøj VG, Dueholm M, Mamsen LS, Ernst E, Andersen CY. Hormonal response in patients transplanted with cryopreserved ovarian tissue is independent of whether freezing was performed in childhood or adulthood. J Assist Reprod Genet. 2021 Nov 1;38(11):3039–45. doi:10.1007/s10815-021-02320-z PubMed PMID: 34617199.

26.	Anderson RA, McLaughlin M, Wallace WHB, Albertini DF, Telfer EE. The immature human ovary shows loss of abnormal follicles and increasing follicle developmental competence through childhood and adolescence. Human Reproduction. 2014;29(1):97–106. doi:10.1093/humrep/det388 PubMed PMID: 24135076.

27.	Imbert R, Moffa F, Tsepelidis S, Simon P, Delbaere A, Devreker F, et al. Safety and usefulness of cryopreservation of ovarian tissue to preserve fertility: A 12-year retrospective analysis. Human Reproduction. 2014;29(9):1931–40. doi:10.1093/humrep/deu158 PubMed PMID: 24958067.

28.	Wallace WHB, Smith AG, Kelsey TW, Edgar AE, Anderson RA. Fertility preservation for girls and young women with cancer: Population-based validation of criteria for ovarian tissue cryopreservation. Lancet Oncol. 2014;15(10):1129–36. doi:10.1016/S1470-2045(14)70334-1 PubMed PMID: 25130994.

29.	Lotz L, Liebenthron J, Nichols-Burns SM, Montag M, Hoffmann I, Beckmann MW, et al. Spontaneous antral follicle formation and metaphase II oocyte from a non-stimulated prepubertal ovarian tissue xenotransplant. Reproductive Biology and Endocrinology. 2014 May 15;12(1). doi:10.1186/1477-7827-12-41 PubMed PMID: 24886634.

30.	Raffel N, Lotz L, Hoffmann I, Liebenthron J, Söder S, Beckmann MW, et al. Repetitive Maturation of Oocytes from Non-Stimulated Xenografted Ovarian Tissue from a Prepubertal Patient Indicating the Independence of Human Ovarian Tissue. Geburtshilfe Frauenheilkd. 2017 Dec 1;77(12):1304–11. doi:10.1055/s-0043-122601

31.	Lima M, Gargano T, Fabbri R, Maffi M, Destro F. Ovarian tissue collection for cryopreservation in pediatric age: Laparoscopic technical tips. J Pediatr Adolesc Gynecol. 2014;27(2):95–7. doi:10.1016/j.jpag.2013.11.002 PubMed PMID: 24602303.

32.	Biasin E, Salvagno F, Berger M, Nesi F, Quarello P, Vassallo E, et al. Ovarian tissue cryopreservation in girls undergoing haematopoietic stem cell transplant: Experience of a single centre. Bone Marrow Transplant. 2015 Sep 4;50(9):1206–11. doi:10.1038/bmt.2015.111 PubMed PMID: 25961773.

33.	Demeestere I, Simon P, Dedeken L, Moffa F, Tsépélidis S, Brachet C, et al. Live birth after autograft of ovarian tissue cryopreserved during childhood. Human Reproduction. 2015 Apr 9;30(9):2107–9. doi:10.1093/humrep/dev128 PubMed PMID: 26062556.

34.	Duncan FE, Pavone ME, Gunn AH, Badawy S, Gracia C, Ginsberg JP, et al. Pediatric and teen ovarian tissue removed for cryopreservation contains follicles irrespective of age, disease diagnosis, treatment history, and specimen processing methods. J Adolesc Young Adult Oncol. 2015 Dec 1;4(4):174–83. doi:10.1089/jayao.2015.0032 PubMed PMID: 26697267.

35.	Segers I, Mateizel I, Van Moer E, Smitz J, Tournaye H, Verheyen G, et al. In vitro maturation (IVM) of oocytes recovered from ovariectomy specimens in the laboratory: a promising “ex vivo” method of oocyte cryopreservation resulting in the first report of an ongoing pregnancy in Europe. J Assist Reprod Genet. 2015 Aug 8;32(8):1221–31. doi:10.1007/s10815-015-0528-9 PubMed PMID: 26253691.

36.	Abir R, Ben-Aharon I, Garor R, Yaniv I, Ash S, Stemmer SM, et al. Cryopreservation of in vitro matured oocytes in addition to ovarian tissue freezing for fertility preservation in paediatric female cancer patients before and after cancer therapy. Human Reproduction. 2016 Apr 1;31(4):750–62. doi:10.1093/humrep/dew007 PubMed PMID: 26848188.

37.	Cai H, Shen H. Random-start controlled ovarian stimulation for emergency fertility preservation in a patient with myelodysplastic syndrome: A case report. Brazilian Journal of Medical and Biological Research. 2016;49(6):1–4. doi:10.1590/1414-431X20165227 PubMed PMID: 27191608.

38.	Chambon F, Brugnon F, Grèze V, Grémeau AS, Pereira B, Déchelotte P, et al. Cryopreservation of ovarian tissue in pediatric patients undergoing sterilizing chemotherapy. Hum Fertil. 2016 Jan 2;19(1):23–31. doi:10.3109/14647273.2016.1151561 PubMed PMID: 27008573.

39.	El Issaoui M, Giorgione V, Mamsen LS, Rechnitzer C, Birkebæk N, Clausen N, et al. Effect of first line cancer treatment on the ovarian reserve and follicular density in girls under the age of 18 years. Fertil Steril. 2016;106(7):1757-1762.e1. doi:10.1016/j.fertnstert.2016.09.001 PubMed PMID: 27717554.

40.	Meirow D, Ra’anani H, Shapira M, Brenghausen M, Derech Chaim S, Aviel-Ronen S, et al. Transplantations of frozen-thawed ovarian tissue demonstrate high reproductive performance and the need to revise restrictive criteria. Fertil Steril. 2016 Aug 1;106(2):467–74. doi:10.1016/j.fertnstert.2016.04.031 PubMed PMID: 27181924.

41.	Lavery SA, Islam R, Hunt J, Carby A, Anderson RA. The medical and ethical challenges of fertility preservation in teenage girls: A case series of sickle cellanaemia patients prior to bonemarrow transplant. Human Reproduction. 2016 Jul 1;31(7):1501–7. doi:10.1093/humrep/dew084 PubMed PMID: 27112701.

42.	Lotz L, Maktabi A, Hoffmann I, Findeklee S, Beckmann MW, Dittrich R. Ovarian tissue cryopreservation and retransplantation - What do patients think about it? Reprod Biomed Online. 2016 Apr 1;32(4):394–400. doi:10.1016/j.rbmo.2015.12.012 PubMed PMID: 26825247.

43.	Van DerVen H, Liebenthron J, Beckmann M, Toth B, Korell M, Krüssel J, et al. Ninety-five orthotopic transplantations in 74 women of ovarian tissue after cytotoxic treatment in a fertility preservation network: Tissue activity, pregnancy and delivery rates. Human Reproduction. 2016 Sep 1;31(9):2031–41. doi:10.1093/humrep/dew165 PubMed PMID: 27378768.

44.	Jadoul P, Guilmain A, Squifflet J, Luyckx M, Votino R, Wyns C, et al. Efficacy of ovarian tissue cryopreservation for fertility preservation: Lessons learned from 545 cases. Human Reproduction. 2017 May 1;32(5):1046–54. doi:10.1093/humrep/dex040 PubMed PMID: 28333228.

45.	Jensen AK, Macklon KT, Fedder J, Ernst E, Humaidan P, Andersen CY. 86 successful births and 9 ongoing pregnancies worldwide in women transplanted with frozen-thawed ovarian tissue: Focus on birth and perinatal outcome in 40 of these children. J Assist Reprod Genet. 2017 Mar 1;34(3):325–36. doi:10.1007/s10815-016-0843-9 PubMed PMID: 28028773.

46.	Tsampras N, Gould D, Fitzgerald CT. Double ovarian stimulation (DuoStim) protocol for fertility preservation in female oncology patients. Hum Fertil. 2017 Oct 2;20(4):248–53. doi:10.1080/14647273.2017.1287433 PubMed PMID: 28423955.

47.	Arian SE, Flyckt RL, Herman R, Erfani H, Falcone T. Fertility preservation in pediatric female cancer patients. Fertil Steril. 2018 May 1;109(5):941. doi:10.1016/j.fertnstert.2018.02.112 PubMed PMID: 29778393.

48.	Kutteh WH, Klosky JL, Green DM, Sparrow CK, Kutteh MA, Robinson GW, et al. Ovulation induction and oocyte retrieval for fertility preservation in young adolescents newly diagnosed with medulloblastoma: a case series. J Obstet Gynaecol (Lahore). 2018 Aug 18;38(6):878–9. doi:10.1080/01443615.2017.1398222 PubMed PMID: 29390921.

49.	Matthews SJ, Picton H, Ernst E, Andersen CY. Successful pregnancy in a women previously suffering from b-thalassemia following transplantation of ovarian tissue cryopreservation before puberty. Minerva Ginecol (2018) 70:432–5. doi: 10.23736/S0026-4784.18.04240-5.

50.	Mamsen LS, Kristensen SG, Pors SE, Bøtkjær JA, Ernst E, Macklon KT, et al. Consequences of β-Thalassemia or Sickle Cell Disease for Ovarian Follicle Number and Morphology in Girls Who Had Ovarian Tissue Cryopreserved. Front Endocrinol (Lausanne). 2021 Jan 15;11. doi:10.3389/fendo.2020.593718

51.	Peddie VL, Maheshwari A. Successful controlled ovarian stimulation and vitrification of oocytes in an adolescent diagnosed with myelodysplastic/pre-malignant clone with monosomy 7. Hum Fertil. 2018;21(1):39–44. doi:10.1080/14647273.2017.1347288 PubMed PMID: 28683583.

52.	Garg D, Johnstone EB, Fair DB, Carrell DT, Berga S, Letourneau JM. Oncofertility conundrum: discrepancy between anti-Mϋllerian hormone and mature oocyte yield in a peripubertal girl with Hodgkin lymphoma. J Assist Reprod Genet. 2019 Aug 15;36(8):1753–6. doi:10.1007/s10815-019-01516-8 PubMed PMID: 31313012.

53.	Hipp HS, Shandley LM, Schirmer DA, McKenzie L, Kawwass JF. Oocyte Cryopreservation in Adolescent Women. J Pediatr Adolesc Gynecol. 2019 Aug 1;32(4):377–82. doi:10.1016/j.jpag.2019.03.001 PubMed PMID: 30904628.

54.	Hoekman EJ, Louwe LA, Rooijers M, van der Westerlaken LAJ, Klijn NF, Pilgram GSK, et al. Ovarian tissue cryopreservation: Low usage rates and high live-birth rate after transplantation. Acta Obstet Gynecol Scand. 2020 Feb 1;99(2):213–21. doi:10.1111/aogs.13735 PubMed PMID: 31538662.

55.	Poirot C, Fortin A, Lacorte JM, Akakpo JP, Genestie C, Vernant JP, et al. Impact of cancer chemotherapy before ovarian cortex cryopreservation on ovarian tissue transplantation. Human Reproduction. 2019 Jun 4;34(6):1083–94. doi:10.1093/humrep/dez047 PubMed PMID: 31116405.

56.	Rodriguez-Wallberg KA, Marklund A, Lundberg F, Wikander I, Milenkovic M, Anastacio A, et al. A prospective study of women and girls undergoing fertility preservation due to oncologic and non-oncologic indications in Sweden–Trends in patients’ choices and benefit of the chosen methods after long-term follow up. Acta Obstet Gynecol Scand. 2019 May 1;98(5):604–15. doi:10.1111/aogs.13559 PubMed PMID: 30723910.

57.	Rowell EE, Corkum KS, Lautz TB, Laronda MM, Walz AL, Madonna MB, et al. Laparoscopic unilateral oophorectomy for ovarian tissue cryopreservation in children. J Pediatr Surg. 2019 Mar 1;54(3):543–9. doi:10.1016/j.jpedsurg.2018.06.005 PubMed PMID: 30782317.

58.	Amir H, Oren A, Frishman EK, Sapir O, Shufaro Y, Anat &, et al. Oocyte retrieval outcomes among adolescent transgender males [Internet]. doi:10.1007/s10815-020-01815-5/Published

59.	Manuel SL, Moravek MB, Confino R, Smith KN, Lawson AK, Klock SC, et al. Ovarian stimulation is a safe and effective fertility preservation option in the adolescent and young adult population. J Assist Reprod Genet. 2020 Mar 1;37(3):699–708. doi:10.1007/s10815-019-01639-y PubMed PMID: 31828481.

60.	Nguyen TYT, Camboni A, Masciangelo R, Donnez J, Dolmans MM. Is ovarian tissue transplantation safe in patients with central nervous system primitive neuroectodermal tumors? J Clin Med. 2020 Dec 1;9(12). doi:10.3390/jcm9124101

61.	Hanfling SN, Parikh T, Mayhew A, Robinson E, Graham J, Gomez-Lobo V, et al. Case report: two cases of mature oocytes found in prepubertal girls during ovarian tissue cryopreservation. F and S Reports. 2021 Sep 1;2(3):296–9. doi:10.1016/j.xfre.2021.03.007

62.	Rozen G, Avagliano S, Agresta F, Gook D, Polyakov A, Stern C. Ovarian tissue grafting: Lessons learnt from our experience with 55 grafts. Reprod Med Biol. 2021 Jul 1;20(3):277–88. doi:10.1002/rmb2.12380

63.	Rodriguez-Wallberg KA, Milenkovic M, Papaikonomou K, Keros V, Gustafsson B, Sergouniotis F, et al. Successful pregnancies after transplantation of ovarian tissue retrieved and cryopreserved at time of childhood acute lymphoblastic leukemia - A case report. Haematologica. Ferrata Storti Foundation; 2021. p. 2783–7. doi:10.3324/haematol.2021.278828 PubMed PMID: 34233451.

64.	Fabbri R, Vicenti R, Magnani V, Paradisi R, Lima M, De Meis L, et al. Ovarian tissue cryopreservation and transplantation: 20 years experience in Bologna University. Front Endocrinol (Lausanne). 2022 Oct 12;13. doi:10.3389/fendo.2022.1035109

65.	Ruan X, Cheng J, Du J, Jin F, Gu M, Li Y, et al. Analysis of Fertility Preservation by Ovarian Tissue Cryopreservation in Pediatric Children in China. Front Endocrinol (Lausanne). 2022;13(June):1–10. doi:10.3389/fendo.2022.930786

66.	McElhinney KL, Kennedy T, Rowell EE, Laronda MM. A dozen years of ovarian tissue cryopreservation at a pediatric hospital: tracking program and patient metrics while adapting to increasing needs. F and S Reports. 2024 Jun 1;5(2):197–204. doi:10.1016/j.xfre.2024.02.009

67.	Yahyaei A, Moridi M, Ghaffari F. CASE REPORT Open Access Controlled ovarian stimulation in cancer patients under 18 years old; a case series. J Ovarian Res. 2024;17:33. doi:10.1186/s13048-024-01352-0

68.	Emrich NLA, Einenkel R, Färber CM, Schallmoser A, Sänger N. Ovarian tissue cryopreservation for fertility preservation: a two-decade single-center experience with 451 children and adolescents. Reproductive Biology and Endocrinology. 2025 Dec 1;23(1). doi:10.1186/s12958-025-01388-x PubMed PMID: 40188142.

69.	Latif S, Davies M, Vaughan E, Mavrelos D, Lavery S, Yasmin E. Clinical and ethical perspectives of ovarian stimulation and oocyte cryopreservation in adolescents: 6 years experience from a tertiary centre. Hum Reprod Open. 2025;2025(1). doi:10.1093/hropen/hoaf005

70.	Fee N, Joyce N, Horan M, Glover L, Crosby D. Outcomes of adolescents and young adults (AYA) attending for fertility counselling and oocyte cryopreservation prior to gonadotoxic treatment. J Assist Reprod Genet. 2026. doi:10.1007/s10815-026-03889-z PubMed PMID: 42068486.

71.	Dolmans MM, Donnez J. Fertility preservation in women for medical and social reasons: Oocytes vs ovarian tissue. Best Pract Res Clin Obstet Gynaecol. 2021;70(xxxx):63–80. doi:10.1016/j.bpobgyn.2020.06.011 PubMed PMID: 32800711.

72.	Dunlop C, Jack S, Telfer E, Zahra S, Anderson R. Clinical pregnancy in Turner syndrome following re-implantation of cryopreserved ovarian cortex. J Assist Reprod Genet. 2023;40(10):2385–90. doi:10.1007/s10815-023-02905-w PubMed PMID: 37566317.

73.	Rodriguez-Wallberg KA, Sergouniotis F, Nilsson HP, Lundberg FE. Trends and outcomes of fertility preservation for girls, adolescents and young adults with Turner syndrome: A prospective cohort study. Front Endocrinol (Lausanne). 2023;14(March):1–8. doi:10.3389/fendo.2023.1135249
